# Genomic Analysis of Carbapenem-Resistant *Acinetobacter baumannii* Isolates Belonging to Major Endemic Clones in South America

**DOI:** 10.3389/fmicb.2020.584603

**Published:** 2020-11-30

**Authors:** Carolina Silva Nodari, Rodrigo Cayô, Ana Paula Streling, Felipe Lei, Julia Wille, Myriam S. Almeida, Alexandre Inacio de Paula, Antonio Carlos Campos Pignatari, Harald Seifert, Paul G. Higgins, Ana Cristina Gales

**Affiliations:** ^1^Universidade Federal de São Paulo (UNIFESP), Laboratório Alerta, Division of Infectious Diseases, Department of Internal Medicine, Escola Paulista de Medicina (EPM), São Paulo, Brazil; ^2^Universidade Federal de São Paulo (UNIFESP), Laboratório de Imunologia e Bacteriologia (LIB), Setor de Biologia Molecular, Microbiologia e Imunologia – Departamento de Ciências Biológicas (DCB), Instituto de Ciências Ambientais, Químicas e Farmacêuticas (ICAQF), Diadema, Brazil; ^3^Institute for Medical Microbiology, Immunology and Hygiene, University of Cologne, Cologne, Germany; ^4^German Center for Infection Research (DZIF), Partner Site Bonn-Cologne, Cologne, Germany; ^5^Laboratório de Microbiologia, Hospital Universitário Cassiano Antônio de Moraes, Universidade Federal do Espírito Santo (UFES), Vitória, Brazil; ^6^Setor de Microbiologia – Serviço de Análises Clínicas, Hospital do Servidor Público Estadual (IAMSPE), São Paulo, Brazil; ^7^Universidade Federal de São Paulo (UNIFESP), Laboratório Especial de Microbiologia Clínica (LEMC), Division of Infectious Diseases, Department of Internal Medicine, Escola Paulista de Medicina (EPM), São Paulo, Brazil

**Keywords:** Brazil, IC4, IC5, CHDL, outer membrane proteins, polymyxin resistance, nosocomial infection, two component systems

## Abstract

Carbapenem-resistant *Acinetobacter baumannii* (CRAB) are emerging worldwide. In South America, clinical isolates presenting such a phenotype usually do not belong to the globally distributed international clone 2 (IC2). The majority of these isolates are also resistant to multiple other antimicrobials and are often designated extremely drug-resistant (XDR). The aim of this study was to characterize the resistance mechanisms presented by 18 carbapenem-resistant *A. baumannii* isolates from five different Brazilian hospitals. Species identification was determined by *rpoB* sequencing, and antimicrobial susceptibility was determined by broth microdilution. Isolates were submitted to whole genome sequencing using Illumina platform and genetic similarity was determined by PFGE, MLST, and cgMLST. Genome analysis was used to identify intrinsic and acquired resistance determinants, including mutations in the AdeRSABC efflux system and in outer membrane proteins (OMPs). All isolates were identified as *A. baumannii* and grouped into 4 pulsotypes by PFGE, which belonged to clonal complexes (CC) 15^*Pas*^/103^*Ox*^ (*n* = 4) and 79^*Pas*^/113^*Ox*^ (*n* = 14), corresponding to IC4 and IC5, respectively. High MIC values to carbapenems, broad-spectrum cephalosporins, amikacin, and ciprofloxacin were observed in all isolates, while MICs of ampicillin/sulbactam, gentamicin, and tigecycline varied among the isolates. Minocycline was the most active antimicrobial agent tested. Moreover, 12 isolates (66.7%) were considered resistant to polymyxins. Besides intrinsic OXA-51 and ADC variants, all isolates harbored an acquired carbapenem-hydrolyzing class D β-lactamase (CHDL) encoding gene, either *bla*_*OXA–*__23_ or *bla*_*OXA–*__72_. A diversity of aminoglycoside modifying enzymes and resistance determinants to other antimicrobial classes were found, as well as mutations in *gyrA* and *parC*. Non-synonymous mutations have also been identified in the AdeRSABC efflux system and in most OMPs, but they were considered natural polymorphisms. Moreover, resistance to polymyxins among isolates belonging to IC5 were associated to non-synonymous mutations in *pmrB*, but no known polymyxin resistance mechanism was identified in isolates belonging to IC4. In conclusion, *A. baumannii* clinical isolates belonging to South America’s major clones present a myriad of antimicrobial resistance determinants. Special attention should be paid to natural polymorphisms observed in each clonal lineage, especially regarding non-synonymous mutations in constitutive genes associated with distinct resistance phenotypes.

## Introduction

*Acinetobacter baumannii* is a major nosocomial pathogen causing serious infections (Antunes et al., 2014). In Brazil, it is the fourth most frequent pathogen recovered from central catheter-associated bloodstream infections (BSI) in adult intensive care units (ICU), with carbapenem resistance rates as high as 79% (Agência Nacional de Vigilância Sanitária [ANVISA], 2020). Carbapenem resistance in *A. baumannii* is mainly caused by horizontal transfer of carbapenem-hydrolyzing class D β-lactamases (CHDL) encoding genes, particularly in worldwide epidemic clones (Higgins et al., 2010).

Most carbapenem-resistant isolates also harbor resistance determinants to other antimicrobial classes, such as aminoglycosides and fluoroquinolones. Additionally, resistance to polymyxins has been sporadically reported among some carbapenem-resistant endemic clones (Qureshi et al., 2015), and these isolates are often classified as XDR or pan-drug resistant (PDR) (Nowak et al., 2017). Carbapenem-resistant *A. baumannii* (CRAB) isolates usually belong to the worldwide disseminated international clone 2 (IC2) (Hamidian and Nigro, 2019) and carbapenem-resistance rates vary between 40 and 80% (Kuo et al., 2012; Chmielarczyk et al., 2016). In Latin America, the frequency of XDR *A. baumannii* has increased from 17 to 86.6% between 1997 and 2016 (Gales et al., 2019).

Interestingly, CRAB isolates in South America are not associated with IC2. In Brazil, as well as Argentina, Chile, and Paraguay, the major carbapenemase-producing clones belong to clonal complexes (CCs) 15 and 79, which correspond to IC4 and IC5, respectively (Higgins et al., 2010; Chagas et al., 2014; Cardoso et al., 2016; Rodríguez et al., 2016; Opazo-Capurro et al., 2019). Despite the variety of studies focusing on the genetic context of carbapenemase encoding genes (Chagas et al., 2017; Romanin et al., 2019), comprehensive studies on the genetic basis of XDR phenotype among isolates representing these important ICs are still missing (Graña-Miraglia et al., 2020). Herein, we explored the molecular determinants associated with resistance toward multiple drugs among *A. baumannii* clinical isolates belonging to the major South American clonal lineages recovered in distinct Brazilian hospitals.

## Materials and Methods

### Bacterial Strains and Antimicrobial Susceptibility Testing

A total of 18 *Acinetobacter* spp. clinical isolates were included in study. They were recovered in five tertiary hospitals located in two Brazilian states between April 2012 and October 2017. Isolates were identified to the species level by *rpoB* sequencing as previously described (La Scola et al., 2006). Minimal Inhibitory Concentrations (MICs) for amikacin, ceftazidime, ciprofloxacin, colistin, cefepime, gentamicin, imipenem, meropenem, minocycline, polymyxin B, and tigecycline (Sigma-Aldrich, St. Louis, United States) were determined by cation-adjusted broth microdilution and interpreted according to Brazilian Committee on Antimicrobial Susceptibility Testing (BrCAST/EUCAST) guidelines^1^, when clinical breakpoints for *Acinetobacter* spp. were available. Ampicillin/sulbactam MICs were determined following the guidelines established by BrCAST/EUCAST for Enterobacteriales.

### Whole Genome Sequencing (WGS) and Draft Genome Analysis

All the isolates were subjected to WGS using an Illumina MiSeq sequencer (Illumina Inc., CA, United States) (Higgins et al., 2017), and genomes were assembled with the program Velvet as part of the SeqSphere v.7.0.4 software (Ridom GmbH, Münster, Germany) as described previously (Higgins et al., 2017). To determine differences in specific ORFs in each draft genome sequence, the genome assemblies were aligned to the *A. baumannii* reference strain ACICU and compared to nucleotide and protein sequences of *A. baumannii* ATCC 19606 (accession number NZ_KL810966.1) and ATCC17978 (accession number NZ_CP018664.1).

### Molecular Typing

Clonal relatedness of *A. baumannii* isolates was determined by pulsed-field gel electrophoresis (PFGE) using *Apa*I restriction enzyme (New England BioLabs, Ipswich, MA, United States) and fingerprints were analyzed with previously described criteria (Seifert et al., 2005). WGS data were also used to characterize the isolates by core genome multilocus sequence typing (cgMLST), as previously published (Higgins et al., 2017) and to determine the sequence types (STs) using PubMLST^2^.

### Resistome Analysis

Intrinsic and acquired resistance determinants were screened using ResFinder, version 3.2^3^ and manually curated using Artemis. The presence of insertion sequences upstream of β-lactamase encoding genes was also screened using ISfinder^4^. Non-synonymous mutations in *gyrA* and *parC* were detected comparing the genome sequences with reference *A. baumannii* strain ATCC 19606. Moreover, amino acid substitutions in polymyxin resistance associated systems LpxACD and PmrAB were detected as described by Gerson et al. (2020). This approach was also used to identify potential amino acid substitutions associated with antimicrobial resistance in the efflux pumps system AdeABC, its regulator AdeRS, and in outer membrane proteins (OMPs) CarO, OmpA, OmpW, Omp33-36, and OccAB1. Additionally, the relative expression of *pmrAB* was determined by qRT-PCR using specific primers previously described (Girardello et al., 2017). Assays were performed in triplicate and expression was compared to that of *A. baumannii* ATCC 19606, using *rpoB* as a normalizing gene (Hornsey et al., 2010).

## Results

### *A. baumannii* Clinical Isolates Belonged to South America’s Most Prevalent Clones

**FIGURE 1:**
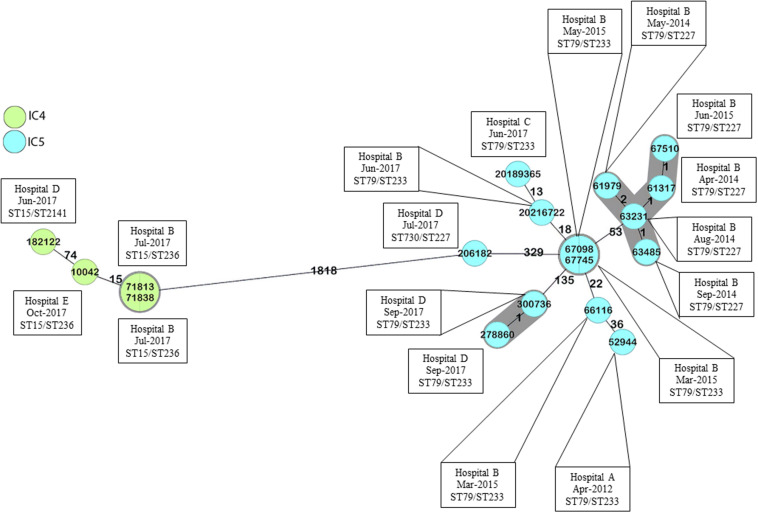
Minimum spanning tree of *A. baumannii* clinical isolates, according to cgMLST. Numbers between isolates indicate the allele differences identified among the 2,390 alleles that belong to *A. baumannii* core genome, ignoring missing values. Colors indicate isolates belonging to the same international clones. Gray zones indicate isolates belonging to the same cluster, and isolate numbers in the same circle were considered identical. Cluster distance threshold: 9. Minimum spanning tree built using Ridom SeqSphere+.

All isolates were identified as *A. baumannii* and were mainly recovered from blood cultures (*n* = 9) followed by lower respiratory tract cultures (*n* = 6) (Table 1). According to PFGE, they were grouped into four distinct pulsotypes, which were included in different STs belonging to Institute Pasteur scheme (Pas) CC79 and CC15, and corresponded to the Oxford scheme (Ox) CC113 and CC103, respectively (Table 1 and Supplementary Figure 1). Interestingly, the ST distribution following the Oxford scheme presented a better correlation to the results observed with PFGE, since ST227, ST233, ST236, and the novel ST2141 were strongly associated with PFGE clusters 1, 2, 4, and 3, respectively (Supplementary Figure 1). Moreover, cgMLST delineated the isolates into four transmission clusters and seven singletons (Figure 1 and Supplementary Figure 1). According to MLST analysis, all CRAB clinical isolates were grouped into two major South American clones, namely IC4 (CC15^*Pas*^/CC103^*Ox*^; *n* = 4) and IC5 (CC79^*Pas*^/CC113^*Ox*^; *n* = 14). It should also be noted that every CC was found over time and in distinct hospitals, confirming the wide spread of those STs in Brazil.

**TABLE 1:** Clinical and epidemiological data and antimicrobial susceptibility profile of *A. baumannii* clinical isolates included in the study.

Isolate	Hospital	State	Collection Date	Clinical Sample	PFGE	ST (Pas/Ox)	MIC (μ g/mL)^*a*^											
							SAM^*b*^	CAZ^*b*^	FEP^*b*^	IPM	MEM	GEN	AMK	CIP	MIN^*b*^	TGC^*b*^	PMB^*c*^	CST^*c*^
52944	A	SP	Apr/13/2012	Blood	1A	79/233	>256/4	>128	128	128	256	8	512	>64	2	16	64	128
61317	B	SP	Apr/11/2014	Urine	1B	79/227	256/4	>128	128	64	64	>128	256	>64	0.5	8	64	>128
61979	B	SP	May/29/2014	Tracheal aspirate	1B	79/227	128/4	>128	128	64	64	>128	128	>64	0.5	8	64	>128
63231	B	SP	Aug/30/2014	Blood	1C	79/227	>256/4	>128	64	128	256	>128	64	>64	≤0.25	0.5	1	1
63485	B	SP	Sep/14/2014	Blood	1C	79/227	>256/4	>128	64	128	128	>128	64	>64	≤0.25	0.5	1	1
66116	B	SP	Mar/25/2015	Tracheal aspirate	2A	79/233	≤0.5/4	>128	128	128	128	4	64	>64	≤0.25	4	16	64
67098	B	SP	May/25/2015	Blood	2B	79/233	>256/4	>128	32	128	256	8	64	>64	0.5	0.5	1	1
67510	B	SP	Jun/22/2015	Tracheal aspirate	1B	79/227	256/4	>128	256	64	64	>128	128	>64	0.5	8	64	>128
67745	B	SP	Jul/14/2015	Blood	2C	79/233	>256/4	>128	128	128	256	16	64	>64	0.5	1	1	1
20189365	C	SP	Jun/03/2017	Tracheal aspirate	2D	79/233	≤0.5/4	>128	64	128	64	4	128	>64	1	8	4	16
20216722	B	SP	Jun/09/2017	Tracheal aspirate	2D	79/233	≤0.5/4	>128	128	256	256	8	128	>64	0.5	4	8	16
182122	D	SP	Jun/19/2017	CSF	3	15/2141	≤0.5/4	8	64	128	32	≤0.5	16	>64	0.5	16	32	>128
71838	B	SP	Jul/05/2017	Blood	4A	15/236	>256/4	>128	64	128	128	2	32	32	≤0.25	0.5	≤0.25	≤0.25
206182	D	SP	Jul/10/2017	Ascitic fluid	2E	730/227	256/4	128	>256	>256	256	>128	256	>64	1	16	8	16
71813	B	SP	Jul/14/2017	Blood	4B	15/236	>256/4	>128	128	256	256	4	64	>64	≤0.25	0.5	≤0.25	≤0.25
278860	D	SP	Sep/11/2017	Blood	2F	79/233	>256/4	>128	>256	>256	128	4	128	>64	1	8	64	>128
300736	D	SP	Sep/28/2017	BAL	2F	79/233	>256/4	>128	>256	>256	128	16	128	>64	1	16	16	64
10042	E	ES	Oct/06/2017	Catheter blood	4C	15/236	>256/4	>128	128	64	128	>128	64	>64	0.5	4	64	>128

*AMK, amikacin; BAL, bronchoalveolar lavage; CAZ, ceftazidime; CIP, ciprofloxacin; CSF, cerebrospinal fluid; CST, colistin; ES, Espírito Santo; FEP, cefepime; GEN, gentamicin; IPM, imipenem; MEM, meropenem; MIC, minimal inhibitory concentration; MIN, minocycline; Ox, Oxford scheme; Pas, Pasteur scheme; PFGE, pulsed field gel electrophoresis; PMB, polymyxin B; SAM, ampicillin/sulbactam; SP, São Paulo; ST, sequence type; TGC, tigecycline. ^*a*^*In vitro* susceptibility according to BrCAST/EUCAST clinical breakpoints are highlighted in the gray boxes. ^*b*^Clinical breakpoints not established by BrCAST/EUCAST. ^*c*^BrCAST provided breakpoints for both polymyxins, while EUCAST provided only for colistin.*

### Most *A. baumannii* Were Highly Resistant to Diverse Antimicrobials

All isolates were resistant to a broad range of antimicrobial agents (Table 1). High MICs were observed in most isolates for ampicillin/sulbactam (MIC range, ≤0.5–>256/4 μg/mL), ceftazidime (MIC range, 8–>128 μg/mL), cefepime (32–>256 μg/mL), imipenem (64–>256 μg/mL), meropenem (32–256 μg/mL), amikacin (16–512 μg/mL), and ciprofloxacin (32–>64 μg/mL). Additionally, 66 and 72% of the isolates were resistant to gentamicin (≤0.5–>128 μg/mL) and both polymyxins (polymyxin B and colistin; ≤0.25–>128 μg/mL), respectively. On the other hand, MICs values for tigecycline ranged from 0.5 to 16 μg/mL. Only minocycline showed consistent activity against all *A. baumannii* isolates (MICs, ≤0.25–2 μg/mL; Table 1). Interestingly, polymyxin B presented a higher *in vitro* activity compared to colistin against polymyxin-resistant isolates, since colistin MICs were at least two-fold dilutions higher than polymyxin B. In contrast, polymyxin B and colistin MICs did not differ among polymyxin-susceptible isolates.

### A Variety of Intrinsic and Acquired β-Lactamases Were Found in CRAB Isolates, Including Novel Variants of *bla*_*ADC*_

**FIGURE 2:**
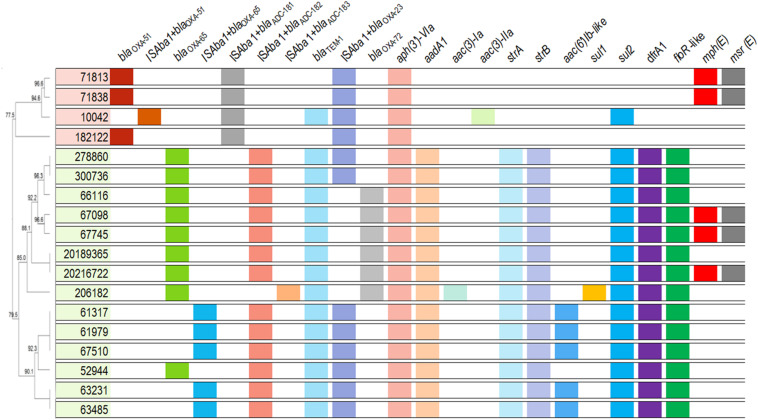
Overview of the acquired antimicrobial resistance determinants and intrinsic β-lactamases encoding genes observed in *A. baumannii* clinical isolates. Isolates belonging to distinct CCs are indicated by different colors. Dendrogram was generated using UPGMA method with BioNumerics, version 7.5, based on ApaI-PFGE results.

As expected, all *A. baumannii* isolates harbored intrinsic chromosome encoded *bla*_*OXA–*__51__–like_ genes. Moreover, each variant was correlated to an IC, being *bla*_*OXA–*__65_ (*n* = 14; IC5, CC79^*Pas*^) the most frequently identified (Figure 2 and Supplementary Table 1). The insertion sequence (IS) IS*Aba1* was found upstream of those genes in one and five isolates belonging to IC4 and IC5, respectively. All isolates harbored novel variants of *bla*_*ADC*_ flanked upstream by IS*Aba1*, namely *bla*_*ADC–*__181_ (*n* = 4), *bla*_*ADC–*__182_ (*n* = 13), and *bla*_*ADC–*__183_ (*n* = 1), and were associated with ST15^*Pas*^, ST79^*Pas*^, and ST730^*Pas*^, respectively. The acquired CHDL encoding genes *bla*_*OXA–*__23_ and *bla*_*OXA–*__72_ were found in 13 (72.2%) and five (27.8%) *A. baumannii* isolates, respectively. The *bla*_*OXA–*__23_ gene was also flanked upstream by IS*Aba1* in all isolates included in the study. Interestingly, *bla*_*OXA–*__72_ was only detected in isolates belonging to IC5 (Figure 2 and Supplementary Table 1). Furthermore, the narrow-spectrum β-lactamase encoding gene *bla*_*TEM–*__1_ was also found in 83.3% of isolates (*n* = 15). No other β-lactamase encoding gene was detected among these isolates.

### Each IC Was Associated With Distinct Resistance Determinants

The reduced susceptibility to amikacin observed in all isolates was associated with the presence of the aminoglycoside modifying enzyme (AME) *aph(3′)-VIa* (Figure 2 and Supplementary Table 1). Additionally, the isolates belonging to IC5 also harbored the phosphotransferase encoding genes *strA* and *strB*, as well as the nucleotidyltransferase encoding gene *aadA1*. Moreover, one IC5 genetic cluster produced an acetyltransferase belonging to the AAC(6′)-Ib family, which explains the high gentamicin resistance levels observed in such isolates (>128 μg/mL). On the other hand, only one isolate belonging to IC4 harbored an acetyltransferase encoding gene, *aac(3)-IIa*, and no additional AME was observed in IC4 isolates (Figure 2 and Supplementary Table 1). The arsenal of acquired resistance determinants observed in IC5 also included *sul2*, *dfrA1*, and *floR*-like genes, which promote reduced susceptibility to sulfamethoxazole, trimethoprim, and chloramphenicol, respectively (Figure 2 and Supplementary Table 1). Additionally, the ST79^*Pas*^ isolates 67098, 67745, and 20216722 harbored the macrolide resistance determinants *mph(E)* and *msr(E)*, which were also found in the ST15^*Pas*^ isolates 71813 and 71838. Finally, the isolate 206182 harbored both *sul1* and *sul2*.

### Resistance to Fluoroquinolones and Polymyxins Was Associated With Point Mutations in Constitutive Genes

**FIGURE 3:**
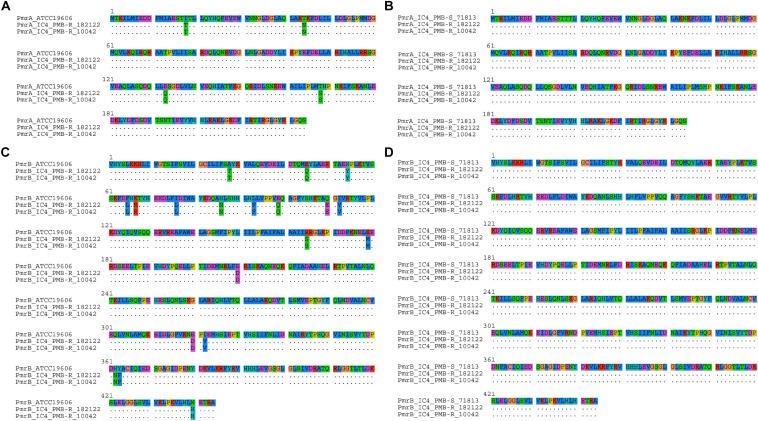
Alignment of PmrA **(A,B)** and PmrB **(C,D)** protein sequences observed in polymyxin-resistant *A. baumannii* clinical isolates 182122 and 10042 belonging to IC4, compared to *A. baumannii* reference strain ATCC 19606 **(A,C)** or to the polymyxin-susceptible IC4 *A. baumannii* isolate 71813 **(B,D)**.

The high ciprofloxacin MICs observed in all the isolates can be explained by the simultaneous presence of Ser_83_Leu and Ser_80_Leu amino acid substitutions in the quinolone resistance determinant region (QRDR) of *gyrA* and *parC*, respectively (Supplementary Table 1). Additionally, point mutations were observed in *pmrAB* in most polymyxin-resistant isolates belonging to IC5 (Table 2). However, the presence of such mutations was not always associated with overexpression of that two-component system (TCS) (Supplementary Figure 2). Interestingly, IS*Aba125* was observed within *pmrA* in two polymyxin-susceptible *A. baumannii* isolates belonging to ST79^*Pas*^, which might have disrupted the function of this transcriptional regulator (Supplementary Table 1). In fact, these isolates presented a reduced expression of *pmrA*, as assessed by qRT-PCR (Supplementary Figure 2). It was also worth noting that some mutations observed in IC5 were present in both polymyxin-resistant and -susceptible isolates and were considered natural polymorphisms associated with this IC, including a duplication of ten amino acids in PmrB observed in some isolates (Supplementary Table 1). A high number of polymorphisms was also observed in isolates belonging to IC4 (Figure 3). However, none of them was exclusively found in polymyxin-resistant isolates, suggesting that such substitutions were not associated with polymyxin resistance in IC4, and might explain the absence of *pmrAB* overexpression among those isolates (Supplementary Table 1 and Supplementary Figure 2).

**TABLE 2:** Amino acid substitutions and relative expression of TCS PmrAB in polymyxin-resistant *A. baumannii* clinical isolates belonging to IC5.

Isolate	ST (Pas/Ox)	PmrA		PmrB	
		Amino acid substitution^*a*^	Relative expression^*b*^	Amino acid substitution^*a*^	Relative expression^*b*^
52944	79/233	ND	2.37	ND	2.17
61317	79/227	ND	4.45	ND	6.61
61979	79/227	ND	9.16	ND	8.43
66116	79/233	ND	−3.36	Tyr_149_Phe, Gln_240_Glu	−5.81
67510	79/227	ND	3.64	ND	5.37
20189365	79/233	Asp_10_Val	−2.9	Gly_414_Arg	1.13
20216722	79/233	ND	2.67	Arg_263_Cys	−4.17
206182	730/227	ND	1.15	Ser_14_Ala	−4.08
278860	79/233	ND	2.58	Thr_187_Phe, Leu_272_Phe	1.99
300736	79/233	ND	5.52	Thr_187_Phe, Leu_272_Phe	3.11

*ND, not detected; Ox, Oxford scheme; Pas, Pasteur scheme; ST, sequence type. ^*a*^Amino acid substitutions were identified based on the comparison with polymyxin-susceptible isolates belonging to IC5. ^*b*^Relative expression of *pmrAB* is expressed in terms of fold change, compared to transcriptional levels observed in *A. baumannii* ATCC 19606.*

The substitutions observed in LpxD in all isolates were also considered to be natural polymorphisms, as they were strictly related to the genetic background of the isolates rather than their resistance phenotype (Supplementary Table 1). While only isolates belonging to IC5 harbored the *pmrC* homolog *eptA*, no *mcr* genes were detected among the 18 *A. baumannii* isolates evaluated. In fact, no known polymyxin resistance mechanism was identified among those isolates belonging to IC4, suggesting that novel mechanisms might be responsible for the high polymyxins MICs (ranging from 32 to >128 μg/mL) observed in isolates 182122 and 10042 (Table 1).

### OMPs and AdeA Protein Sequences Were Distinct in Each Endemic Clone

**FIGURE 4:**
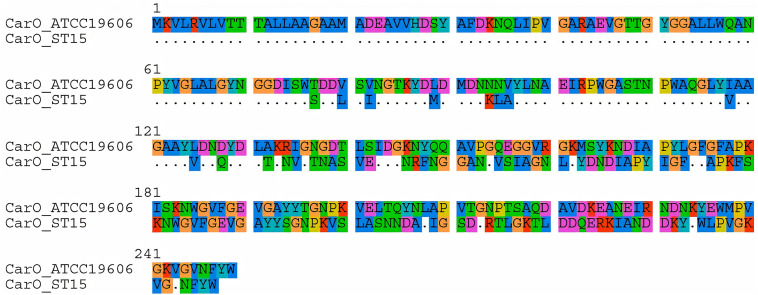
Alignment of CarO protein sequences observed in *A. baumannii* clinical isolates belonging to IC4, compared to *A. baumannii* reference strain ATCC 19606.

Genes encoding OMPs also presented clone-associated alleles. As shown in Table 3, the allelic variation of *ompA* included non-synonymous mutations specific for each CC. This was also observed in CarO, where isolates belonging to IC5 presented protein sequences identical to the one observed in the *A. baumannii* reference strain ATCC 19606, while those belonging to IC4 presented more than 60 amino acid substitutions (73.1% identity; Figure 4 and Supplementary Table 1). In contrast, the OprD-like protein OccAb1 was identical in both ICs, even though three amino acid substitutions were observed compared to the reference strain (Table 3). No amino acid substitutions were observed in OmpW and Omp33-36.

**TABLE 3:** Amino acid substitutions observed in OMPs in *A. baumannii* clinical isolates.

Isolate	OmpA^*a*^	OmpW^*a*^	OccAb1^*a*^	Omp33-36^*a*^
52944	Gly_52_Ser, Thr_144_Asn	WT	Ile_121_Leu, Leu_293_Ile, Arg_445_His	WT
61317	Gly_52_Ser, Thr_144_Asn	WT	Ile_121_Leu, Leu_293_Ile, Arg_445_His	WT
61979	Gly_52_Ser, Thr_144_Asn	WT	Ile_121_Leu, Leu_293_Ile, Arg_445_His	WT
63231	Gly_52_Ser, Thr_144_Asn	WT	Ile_121_Leu, Leu_293_Ile, Arg_445_His	WT
63485	Gly_52_Ser, Thr_144_Asn	WT	Ile_121_Leu, Leu_293_Ile, Arg_445_His	WT
66116	Gly_52_Ser, Thr_144_Asn	WT	Ile_121_Leu, Leu_293_Ile, Arg_445_His	WT
67098	Gly_52_Ser, Thr_144_Asn	WT	Ile_121_Leu, Leu_293_Ile, Arg_445_His	WT
67510	Gly_52_Ser, Thr_144_Asn	WT	Ile_121_Leu, Leu_293_Ile, Arg_445_His	WT
67745	Gly_52_Ser, Thr_144_Asn	WT	Ile_121_Leu, Leu_293_Ile, Arg_445_His	WT
20189365	Gly_52_Ser, Thr_144_Asn	WT	Ile_121_Leu, Leu_293_Ile, Arg_445_His	WT
20216722	Gly_52_Ser, Thr_144_Asn	WT	Ile_121_Leu, Leu_293_Ile, Arg_445_His	WT
182122	Gly_52_Ala	WT	Ile_121_Leu, Leu_293_Ile, Arg_445_His	WT
71838	Gly_52_Ala	WT	Ile_121_Leu, Leu_293_Ile, Arg_445_His	WT
206182	Gly_52_Ser, Thr_144_Asn	WT	Ile_121_Leu, Leu_293_Ile, Arg_445_His	WT
71813	Gly_52_Ala	WT	Ile_121_Leu, Leu_293_Ile, Arg_445_His	WT
278860	Gly_52_Ser, Thr_144_Asn	WT	Ile_121_Leu, Leu_293_Ile, Arg_445_His	WT
300736	Gly_52_Ser, Thr_144_Asn	WT	Ile_121_Leu, Leu_293_Ile, Arg_445_His	WT
10042	Gly_52_Ala	WT	Ile_121_Leu, Leu_293_Ile, Arg_445_His	WT

*OMP, outer membrane protein; WT, wild type. ^*a*^Amino acid substitutions are based on sequences of reference strain *A. baumannii* ATCC 19606.*

**FIGURE 5:**
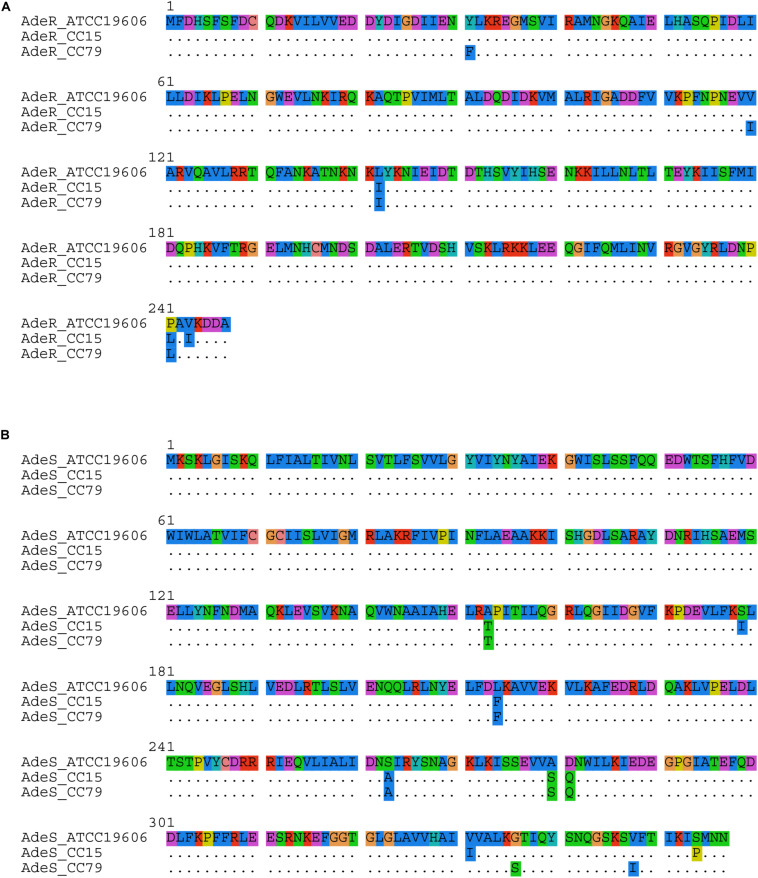
Alignment of **(A)** AdeR and **(B)** AdeS protein sequences observed in *A. baumannii* clinical isolates belonging to IC4 and IC5, compared to *A. baumannii* reference strain ATCC 19606.

Even though the protein sequences of AdeB were conserved in *A. baumannii* clinical isolates belonging to both IC4 and IC5 and identical to the *A. baumannii* reference strain ATCC 19606, *adeA* presented distinct alleles in each IC. While this gene was identified as wild type in isolates belonging to IC5, two amino acid substitutions were identified in all IC4 isolates, namely Ala_368_Leu and Thr_386_Asn (Supplementary Table 1). Moreover, proteins belonging to the TCS AdeRS also presented distinct sequences in each CC, which differ from ATCC 19606 (Figure 5). It should be noted that some amino acid substitutions were observed in both IC4 and IC5 isolates, such as Val_136_Ala and Leu_142_Ile in AdeR, and Ala_153_Thr, Leu_214_Phe, Ser_263_Ala, Ala_280_Ser, and Asp_281_Gln in AdeS.

## Discussion

The worldwide spread of multidrug-resistant (MDR) *A. baumannii* clones is considered a public health concern. The World Health Organization considers research and surveillance of emerging resistance phenotypes as one of the strategic goals of their Global Action Plan on Antimicrobial Resistance (WHO, 2015). Over the years, the evolution of successful *A. baumannii* clones allowed them to acquire an increasing number of resistance determinants, not only via mobile genetic elements, but also through mutations in constitutive genes (Antunes et al., 2014). In the present study we demonstrated that this genetic plasticity is also responsible for resistance to several antimicrobials among South American *A. baumannii* major clones.

The importance of *A. baumannii* clinical isolates belonging to IC4 and IC5 in South America has been previously demonstrated (Cieslinski et al., 2013; Stietz et al., 2013; Rodríguez et al., 2016; Opazo-Capurro et al., 2019; Müller et al., 2019). The higher frequency of those clones was also observed in different regions in Brazil and has been associated with an XDR phenotype (Royer et al., 2018; Pagano et al., 2019; Tavares et al., 2019). Interestingly, despite their wide distribution through time and space, the IC4 and IC5 clinical isolates included in this study remained highly genetically related, as determined using both PFGE and cgMLST. Vasconcelos et al. (2015) have also identified highly similar *A. baumannii* clinical isolates causing BSI in distinct Brazilian medical centers by REP-PCR, and the most frequent clones described also belonged to IC4 and IC5. Even though most isolates belonging to the same pulsotype were not grouped in the same cluster according to cgMLST, the number of allele differences between those isolates were usually lower than the ones observed between isolates that were not considered genetically related according to PFGE. It should be noted, however, that the cgMLST cluster distance threshold was established based on the allele differences and designed for the investigation of *A. baumannii* transmission clusters or outbreaks (Higgins et al., 2017). These values should be interpreted with caution when evaluating isolates broadly distributed over time and space. In the current study, some isolates were separated in time by several years, and by a distance of over 1000 km. Moreover, it should be noted that isolates belonging to the same ST not always form monophyletic groups based on cgMLST, as previously reported (Castillo-Ramírez and Graña-Miraglia, 2019; Gaiarsa et al., 2019). Additionally, the differences observed in isolates belonging to ST227^*Ox*^ and ST233^*Ox*^ (both part of ST79^*Pas*^) might be associated with recombination events in the vicinities of the *gpi* allele, as described by Hamouda et al. (2010).

The variety of intrinsic β-lactamase encoding genes was also related to the clonal groups each isolate was assigned to. As expected, all isolates belonging to IC5 harbored *bla*_*OXA–*__65_, while *bla*_*OXA–*__51_ was present in all isolates belonging to IC4, as previously described (Zander et al., 2012). However, the presence of the IS*Aba1* upstream the *bla*_*OXA–*__51_-like genes was associated to specific pulsotypes within each IC, namely 1B and 1C from IC5, and 3 from IC4. It should be noted that the low frequency of IS*Aba1*-*bla*_*OXA–*__51_-like structure among Brazilian isolates, especially in *bla*_*OXA–*__23_-producing strains, was previously reported (Pagano et al., 2013). On the other hand, this IS was observed upstream of *bla*_*ADC*_-like genes in all isolates and could have been responsible for the high third-generation cephalosporin MIC values observed (Cardoso et al., 2016). It is worth noting that each novel ADC variant was identified in a different ST, even though it is not yet clear if the presence of specific ADCs in *A. baumannii* isolates can also be linked to an IC, as demonstrated for *bla*_*OXA*__–__51_ (Rodríguez-Martínez et al., 2010; Karah et al., 2017).

Although the intrinsic OXA-51 and ADC variants were closely related to each IC included in the study, the acquired CHDL encoding gene *bla*_*OXA–*__23_ was observed in both ICs, as expected. OXA-23 remains the most frequent carbapenem resistance determinant among *A. baumannii* worldwide (Hamidian and Nigro, 2019), and is highly frequent in IC4 and IC5, as previously described (Rodríguez et al., 2016). On the other hand, the production of OXA-72 was restricted to *A. baumannii* clinical isolates belonging CC79^*Pas*^/CC113^*Ox*^. Previous studies have demonstrated the emergence of this CHDL not only within ST79^*Pas*^, but also within its single locus variant (SLV) ST730, in different Brazilian states (Vasconcelos et al., 2015; de Azevedo et al., 2019), as well as among carbapenem-resistant *A. baumannii* belonging to ST15^*Pas*^ (Pagano et al., 2017), but such wide distribution of *bla*_*OXA–*__72_ was not observed among our isolates.

Overall, isolates belonging to IC5 presented a broader spectrum of acquired antimicrobial resistance determinants compared to IC4. The presence of the AME encoding genes *aadA1*, *strA* and *strB*, as well as *dfrA1*, has been previously associated to class 2 integrons in IC5 in different Latin American countries (Martins et al., 2015; Ramírez et al., 2015; Pagano et al., 2019; Romanin et al., 2019). Moreover, *bla*_*TEM–*__1_ was more frequent in this IC and might have contributed to the high ampicillin-sulbactam MICs observed, especially among OXA-23-producing isolates, as previously described (Krizova et al., 2013; Cardoso et al., 2016). Interestingly, all IC4 and IC5 *bla*_*TEM–*__1_-producing isolates also carried *sul2*, suggesting that both antimicrobial resistance determinants could be located in the same mobile genetic element, as previously reported (Hammad et al., 2019).

Despite the differences observed between the major South American *A. baumannii* clones, the high amikacin resistance levels observed in all isolates was associated with the presence of *aph(3′)-VIa*. The Aph(3′)-VIa AME was first described in *A. baumannii* and its activity spectrum includes amikacin (Ramírez and Tolmasky, 2010). Previous studies have also described the high frequency of this AME among *A. baumannii* clinical isolates worldwide, including in Brazil (Aghazadeh et al., 2013; Polotto et al., 2019). Other studies have also highlighted that gentamicin resistance was associated with distinct acetyltransferases, as observed among our isolates, and have suggested that a combination of AMEs could be responsible for that phenotype, as observed in our isolate 206182 (Ballaben et al., 2018). Additionally, Matos et al. (2019) described the presence of *aac(3)-IIa* and *bla*_*TEM–*__1_ in a plasmid of an *A. baumannii* ST15^*Pas*^ clinical isolate, as observed in our 10042 isolate, which also belonged to the same ST. Interestingly, that plasmid also harbored the CHDL encoding gene *bla*_*OXA–*__58_ (Matos et al., 2019), which was not observed in our isolate.

Despite the critical role that acquired resistance determinants play in the acquisition and maintenance of MDR among *A. baumannii* clones, mutations in constitutive genes can also cause high resistance levels to important antimicrobial agents. Holt et al. (2016) have previously suggested that the presence of mutations in *gyrA* and *parC* was one of the features leading to the epidemiological success of IC1, however, this lineage is now rarely found and yet fluoroquinolone resistance remains high. In fact, the combination of Ser_83_Leu and Ser_80_Leu in GyrA and ParC, respectively, was additive (Vila et al., 1997), leading to high ciprofloxacin MICs, as observed among our isolates.

Mutations in the constitutive *pmrAB* genes seemed to be responsible for reduced susceptibility to polymyxins among IC5 isolates. The majority of amino acid substitutions were observed in PmrB and, to the best of our knowledge, most of them have not been described elsewhere. In fact, amino acid substitutions in the histidine kinase of this TCS seem to play an important role in reduced susceptibility to polymyxin in *A. baumannii*, since a high number of non-synonymous mutations in *pmrB* among colistin-resistant isolates was previously highlighted (Olaitan et al., 2014; Poirel et al., 2017; Gerson et al., 2020). The Ser_119_Thr substitution in PmrA, observed in all isolates belonging to IC5, has been previously associated with polymyxin resistance (Arroyo et al., 2011), including in Brazilian isolates (Leite et al., 2019). However, this substitution was also observed among polymyxin-susceptible isolates, suggesting that it might have just a limited role in this phenotype. Moreover, it should be noted that most substitutions described in our study were considered natural polymorphisms associated with distinct clonal linages. These substitutions were also not associated with the higher *in vitro* activity of polymyxin B, compared to colistin, observed among polymyxin-resistant isolates. Such variation might be related to recombination events, as described for other *Acinetobacter* species (Kim and Ko, 2015). It has been previously suggested that polymyxin B and colistin MIC values may be distinct among *A. baumannii* clinical isolates, but the MIC variations were always ± one-fold dilution, and colistin MICs were not consistently higher than polymyxin B (Gales et al., 2001). To test this hypothesis, a larger number of isolates should be evaluated to confirm the distinct activities of both polymyxins among *A. baumannii* isolates presenting reduced susceptibility to those antimicrobial agents.

Gerson et al. (2020) have recently demonstrated that the identification of PmrCAB amino acid substitutions with potential roles in polymyxin resistance should be carried out in isogenic isolates to eliminate natural polymorphisms between lineages. However, the authors have mainly explored that feature among IC2 isolates. We have demonstrated that this is also true for South American major clones, especially among IC4 isolates, where a high number of *pmrAB* mutations was observed but none of them was unique to polymyxin-resistant isolates. This is also valid for the *lpxACD* operon, which has previously been associated with polymyxin resistance in *A. baumannii* (Moffatt et al., 2010). Some of the amino acid substitutions observed in LpxD have been previously described in polymyxin-susceptible and -resistant isolates (Oikonomou et al., 2015), corroborating our findings and confirming they were natural polymorphisms. Additionally, the absence of *eptA* among IC4 belonging isolates observed in our study has been previously described (Gerson et al., 2019) and highlights the presence of unknown polymyxin resistance mechanisms among CC15^*Pas*^/CC103^*Oxf*^ *A. baumannii* clinical isolates.

The reduced susceptibility to tigecycline observed among some *A. baumannii* isolates suggested that the AdeABC efflux system might have been overexpressed, considering that this glycylcycline is a substrate for this system (Lashinsky et al., 2017). The TCS AdeRS regulates its expression and amino acid substitutions in those proteins have been associated with higher tigecycline MICs (Ruzin et al., 2007; Gerson et al., 2018). Although a high number of amino acid substitutions was observed in AdeRS in the isolates included in this study, they did not seem to have influenced tigecycline MICs, since isolates with the same pattern of mutations presented MICs ranging from 0.5 to 16 μg/mL. In fact, the substitution patterns were identical within each IC, suggesting that they were only natural polymorphisms. Therefore, the activity of other efflux systems, such as AdeIJK, might have been responsible for tigecycline MIC variations observed in our study, as previously reported (Damier-Piolle et al., 2008).

The great variety of resistance determinants observed among the isolates in this study might have masked the role of OMPs in our isolates. The high number of amino substitutions observed, especially in CarO, complicates the identification of mutations with a potential role in antimicrobial resistance. Additionally, the fact that the observed mutation patterns were identical in all isolates belonging to the same lineage suggests that they were polymorphisms associated with the evolution of different clonal lineages. Interestingly, Zhu et al. (2019) have previously highlighted the high variability of CarO protein sequences among *A. baumannii* isolates, including the presence of small insertions and deletions, as observed among ST15^*Pas*^ isolates. Moreover, Mussi et al. (2011) have demonstrated that such diversity could be associated with horizontal gene transfer and assertive recombination. Further studies are required to determine the contribution of this variability to antimicrobial resistance.

In summary, we demonstrated that a diversity of antimicrobial resistance determinants was present in the major South American *A. baumannii* clones. We have also provided evidence that attention should be payed to natural polymorphisms when comparing isolates with distinct phenotypes and genetic background, since most constitutive genes associated with antimicrobial resistance presented amino acid substitutions that did not play a role in reduced antimicrobial susceptibility. In addition, the contribution of high frequency of polymorphisms in hot spot genes among endemic clones must be evaluated with caution. Additionally, we suggested that unknown polymyxin resistance mechanisms might be present in *A. baumannii* isolates belonging to IC4. The resistance phenotype exhibited by endemic *A. baumannii* clones was very worrisome and should be carefully studied, considering that the molecular mechanisms involved seem to be, to some extent, lineage-specific.

## Importance

Carbapenem-resistant *Acinetobacter baumannii* (CRAB) have emerged worldwide due to the dissemination of successful clones. Even though international clone 2 (IC2) is the most prevalent in many countries, in Latin America CRAB isolates often belong to IC4 and IC5. The majority of CRAB isolates are also resistant to multiple other antimicrobials and have been classified as extremely drug-resistant (XDR). However, data exploring the molecular mechanisms involved in this phenotype among IC4 and IC5 are still scarce. In this study we presented a comprehensive analysis of genome sequencing data from Brazilian *A. baumannii* belonging to these important clones. We demonstrated that a combination of intrinsic and acquired resistance determinants was responsible for the resistance to several antimicrobial agents among Brazilian endemic clones. We also suggested that an unknown mechanism was responsible for the emergence of polymyxin resistance among IC4 clinical isolates. Finally, we highlighted the importance of comparing isolates with similar genetic background when evaluating mutations in constitutive genes, including efflux systems and outer membrane protein encoding genes.

## Accession Numbers

All raw reads generated were submitted to the Sequencing Read Archive (https://www.ncbi.nlm.nih.gov/sra/) of the National Center for Biotechnology Information (NCBI) under the BioProject number PRJNA632943. The sequences of the novel *bla*_*ADC–181*_, *bla*_*ADC–182*_, and *bla*_*ADC–183*_ genes were deposited in NCBI β-lactamases database under the accession numbers MK248721, MK248722, and MK248723, respectively.

## Data Availability Statement

The datasets presented in this study can be found in online repositories. The names of the repository/repositories and accession number(s) can be found below: https://www.ncbi.nlm.nih.gov/, PRJNA632943; https://www.ncbi.nlm.nih.gov/, MK248721; https://www.ncbi.nlm.nih.gov/, MK248722; https://www.ncbi.nlm.nih.gov/, MK248723.

## Ethics Statement

Ethical approval for this study was obtained from Research Ethics Committee from Federal University of São Paulo – UNIFESP/São Paulo Hospital (Process number: CEP N 4665141216).

## Author Contributions

CN, RC, PH, HS, and AG contributed to the study conception and design. RC and PH supervised the assays. MA, AIdP, and ACCP provided the strains and clinical data. CN, AS, FL, and JW performed the data collection and analysis. CN wrote the first draft of the manuscript and it was edited by RC. PH, HS, and AG reviewed the final draft of manuscript. All authors read and approved the final version of manuscript.

## Conflict of Interest

AG has recently received research funding and/or consultation fees from Bayer, Cristália, InfectoPharm, Eurofarma, MSD, Pfizer and Zambon. The remaining authors declare that the research was conducted in the absence of any commercial or financial relationships that could be construed as a potential conflict of interest.

## Footnotes

1 http://brcast.org.br/

2 https://pubmlst.org/abaumannii/

3 https://cge.cbs.dtu.dk/services/ResFinder/

4 https://isfinder.biotoul.fr/blast.php
