# Supplementary material for: Genomic Analysis of Carbapenem-Resistant Acinetobacter baumannii Isolates Belonging to Major Endemic Clones in South America
Source: Front Microbiol. 2020 Nov 30;11:584603. doi: 10.3389/fmicb.2020.584603 (PMC7734285; doi:10.3389/fmicb.2020.584603)
Supplement: Supplementary file 2 [file Table_2.pdf]

Supplementary Table S2. Whole-genome sequencing and assembly statistics of *A. baumannii* clinical isolates included in the study.

Isolate ID	Avg. Coverage	Read Count	Avg. Read Length (bp)	Assembly Base Count	Contig Count	N50 (bp)	Avg. Contig Length (bp)	Max. Contig Length (bp)	Min. Contig Length (bp)
52944	70	1463594	187	3891392	530	14092	7342	70468	177
61317	95	1691750	219	4051914	380	25362	10662	80799	205
61979	94	1845066	198	3868427	461	19131	8391	70400	192
63231	75	1724710	170	3815419	535	14782	7131	61611	161
63485	97	1917776	196	3851357	498	17254	7733	49801	185
66116	78	1500004	203	3987347	251	40792	15885	134420	193
67098	99	2007834	193	3972821	240	39022	16553	156766	181
67510	109	2194376	194	4028273	457	20511	8814	81834	192
67745	105	1940996	211	3909264	560	15794	6980	63303	201
20189365	99	1845870	210	3894930	534	16364	7293	78534	201
20216722	118	2337936	197	3916738	446	19712	8781	88433	185
182122	103	1887198	213	3795841	281	27542	13508	70036	201
71838	119	2417018	191	3719202	486	14260	7652	57637	181
206182	117	2221686	204	3996890	423	20656	9448	73002	193
71813	93	2084788	173	3748797	390	19198	9612	65033	161
278860	89	1591158	217	3868684	421	18972	9189	72810	205
300736	104	1903622	212	3969551	421	22412	9428	72848	201
10042	69	1496268	180	3863634	391	22219	9881	81949	169

Raw sequence and assembly data retrieved from SeqSphere v.7.0.4 software, before and after *de novo* genome assembly using Velvet.
