# Supplementary figures and images for: Genomic Analysis of Carbapenem-Resistant Acinetobacter baumannii Isolates Belonging to Major Endemic Clones in South America
Source: Front Microbiol. 2020 Nov 30;11:584603. doi: 10.3389/fmicb.2020.584603 (PMC7734285; doi:10.3389/fmicb.2020.584603)

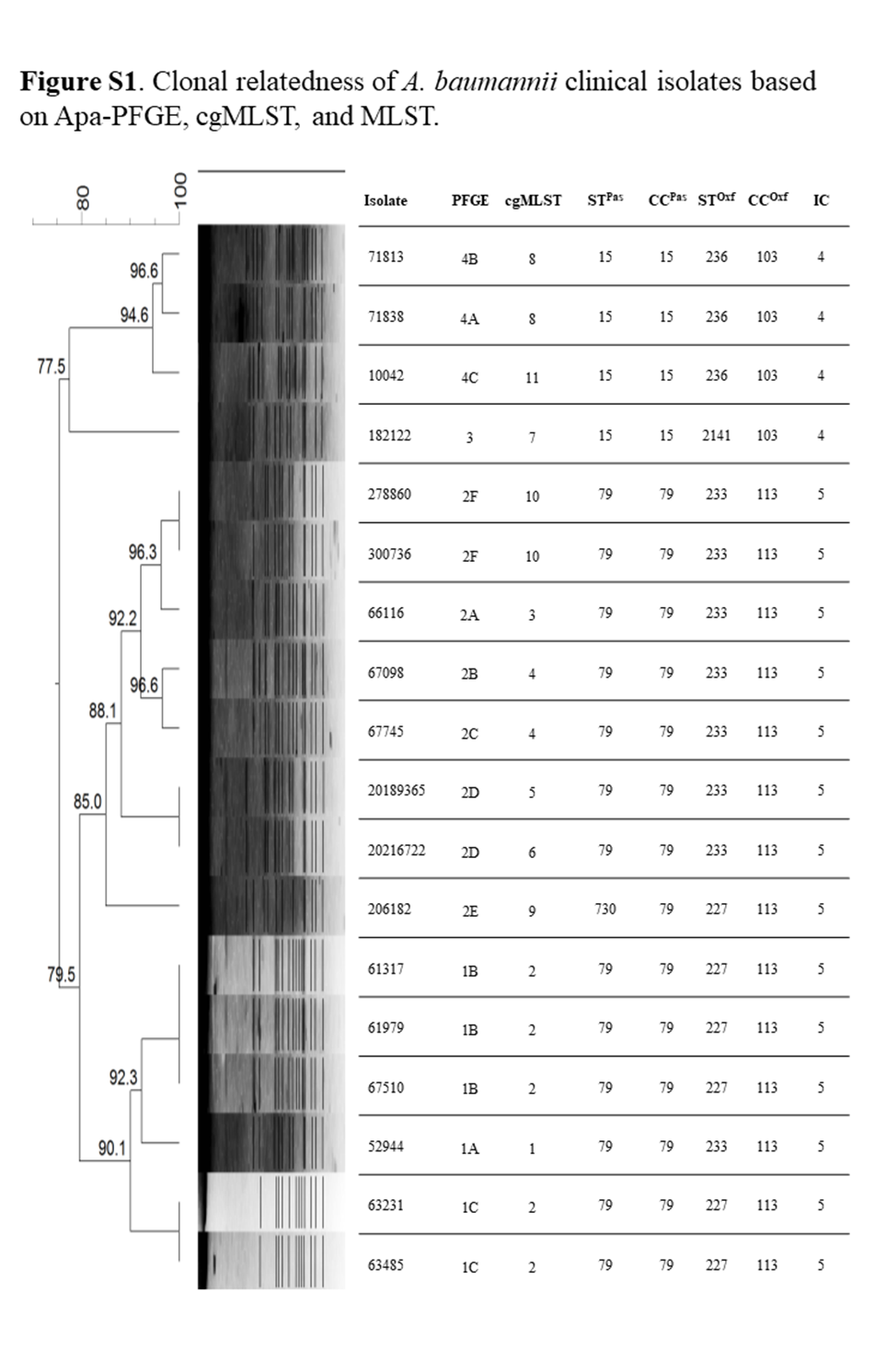

Supplement: Supplementary file 3 [file Image_1.TIF]
