# Supplementary material for: Genomic Analysis of Carbapenem-Resistant Acinetobacter baumannii Isolates Belonging to Major Endemic Clones in South America
Source: Front Microbiol. 2020 Nov 30;11:584603. doi: 10.3389/fmicb.2020.584603 (PMC7734285; doi:10.3389/fmicb.2020.584603)
Supplement: Supplementary file 4 [file Image_2.PDF]

Figure S2. Relative expression of TCS PmrAB in *A. baumannii* clinical isolates

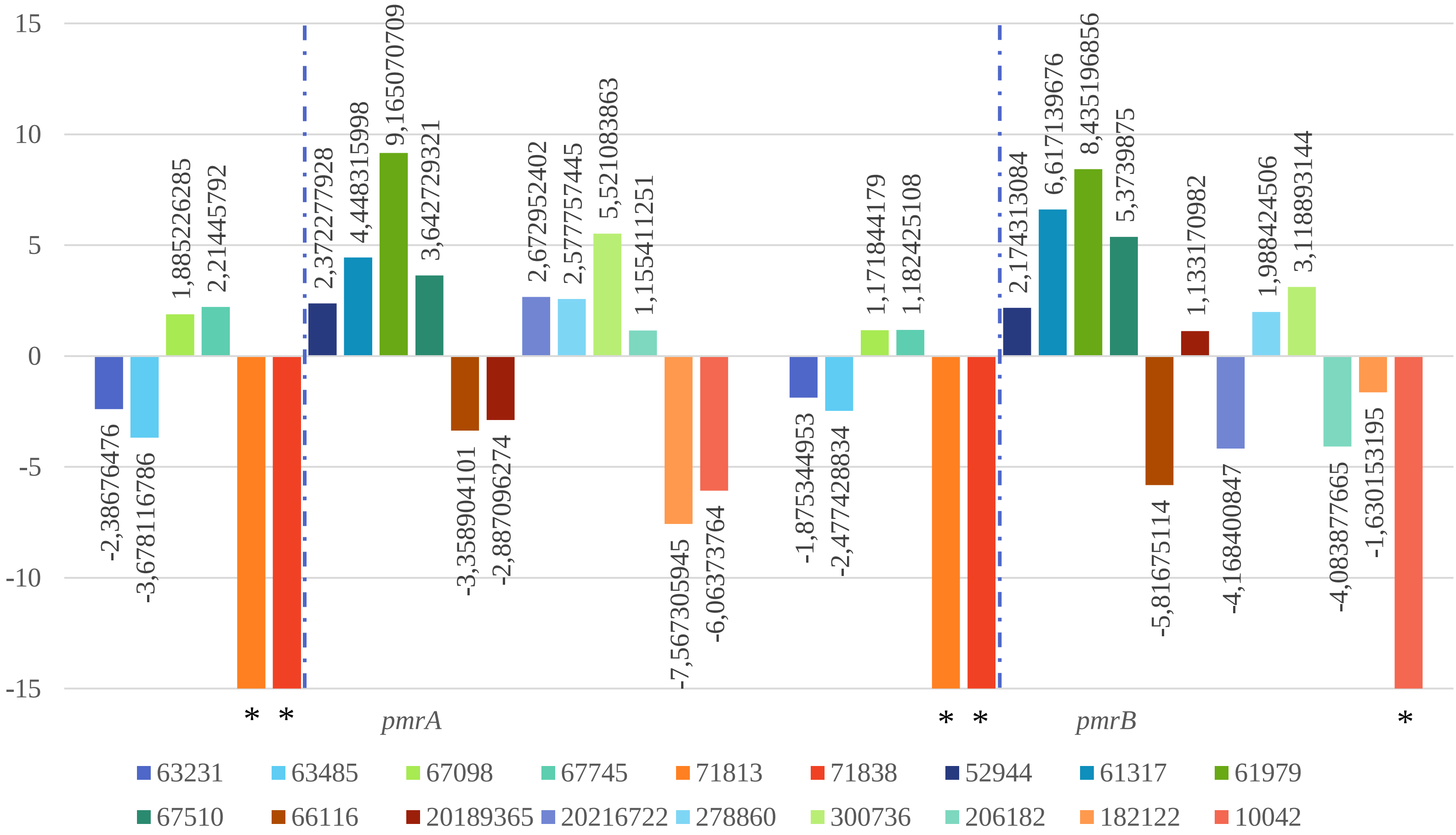

Relative expression of *pmrAB* is expressed in terms of fold change, compared to transcriptional levels observed *A. baumannii* ATCC 19606. Downregulation is expressed as the negative inverse value of the fold change. Dashed lines separate polymyxin-susceptible from –resistant strains. *, expression not detected.
